# Development and Validation of 3 Min Incremental Step-In-Place Test for Predicting Maximal Oxygen Uptake in Home Settings: A Submaximal Exercise Study to Assess Cardiorespiratory Fitness

**DOI:** 10.3390/ijerph182010750

**Published:** 2021-10-13

**Authors:** Fang Li, Chun-Hao Chang, Yu-Chun Chung, Huey-June Wu, Nai-Wen Kan, Wen-Sheng ChangChien, Chin-Shan Ho, Chi-Chang Huang

**Affiliations:** 1 Graduate Institute of Sports Science, National Taiwan Sport University, Taoyuan City 333325, Taiwan; lif848332@gmail.com (F.L.); hao781106@ntsu.edu.tw (C.-H.C.); 2 Center of General Education, Taipei Medical University, Taipei 11031, Taiwan; e750224@tmu.edu.tw (Y.-C.C.); kevinkan@tmu.edu.tw (N.-W.K.); 3 Department of Combat Sports and Chinese Martial Arts, Chinese Culture University, Taipei 11114, Taiwan; wuhc0123@gmail.com; 4 Service Systems Technology Center, Industrial Technology Research Institute, Hsinchu 310401, Taiwan; changchien.ws@gmail.com

**Keywords:** cardiorespiratory fitness, maximal oxygen uptake, 3 min incremental step in place, prediction model

## Abstract

The purpose of this research was to develop the 3 min incremental step-in-place (3MISP) test for predicting maximal oxygen uptake ($\dot{V}$O_2max_). A total of 205 adults (20–64 years) completed the 3MISP and $\dot{V}$O_2max_ tests. Using age, gender, body composition (BC) including percent body fat (PBF) or body mass index (BMI), and with or without heart rate (HR) at the beginning of exercise (HR0) or difference between HR at the third minute during the exercise and the first minute post exercise (ΔHR3 − HR4) in the 3MISP test, six $\dot{V}$O_2max_ prediction models were derived from multiple linear regression. Age (r = −0.239), gender (r = 0.430), BMI (r = −0.191), PBF (r = −0.706), HR0 (r = −0.516), and ΔHR3 − HR4 (r = 0.563) were significantly correlated to $\dot{V}$O_2max_. Among the six $\dot{V}$O_2max_ prediction models, the PBF model^∆HR3 − HR4^ has the highest accuracy. The simplest models with age, gender, and PBF/BMI explained 54.5% of the $\dot{V}$O_2max_ in the PBF model^BC^ and 39.8% of that in the BMI model^BC^. The addition of HR0 and ∆HR3 − HR4 increases the variance of $\dot{V}$O_2max_ explained by the PBF and BMI models^∆HR3 − HR4^ by 17.98% and 45.23%, respectively, while standard errors of estimate decrease by 10.73% and 15.61%. These data demonstrate that the models established using 3MISP-HR data can enhance the accuracy of $\dot{V}$O_2max_ prediction.

## 1. Introduction

In the past, many scholars have devoted themselves to the analysis of aerobic capacity using the submaximal exercise intensity method [1,2,3]. With the public awareness of physical health increasing under the impact of novel coronavirus disease 2019 (COVID-19), at-home workouts have become a pandemic fitness trend. An easy-to-use and low-cost method for self-monitoring of cardiorespiratory fitness (CRF) level is essential for most people. CRF is a primary component of physical fitness [4,5]. The assessment of CRF may help individuals to identify their risk of cardiovascular disease, providing a basis for developing home workout plans, and it may be adopted as a clinical indicator to evaluate a patient’s functional status and treatment outcomes [6,7,8]. Maximal oxygen uptake ($\dot{V}$O_2max_) is a crucial factor in physical performance and health conditions. The plateau in $\dot{V}$O_2_, attained at exhaustion during the incremental $\dot{V}$O_2max_ exercise test, represents the upper limit of CRF [9]. Direct measurement of $\dot{V}$O_2max_ requires the participant to perform an exercise test on a treadmill or bicycle ergometer until exhaustion while being monitored with a gas exchange analyzer. However, this direct measurement of $\dot{V}$O_2max_ relies on highly precise laboratory technology, expensive equipment, complicated operating procedures, intense physical exercise, and appropriate trained personnel to operate the test system. It is also a very time-consuming procedure. In addition, the measurement equipment is stationary, which limits the feasibility of home testing or wide application. Therefore, during the COVID-19 pandemic and increase in home workouts, it is essential to develop a method for indirect measurement of $\dot{V}$O_2max_ suitable for home testing, which could contribute to the development of self-health management in the public.

Exercise tests are important clinical tools for evaluating CRF status and predicting future cardiovascular events [10]. Previous studies have developed various submaximal exercise tests, including the 6 minute walk test, 12 min walk test, Cooper 12 min run test, and the multistage 20 m shuttle run test. Based on the measured distance, speed, and other exercise data, as well as various physiological indicators of body composition, $\dot{V}$O_2max_ prediction equations were established to assess CRF levels in adults [11,12,13]. These exercise tests were relatively simple, required little equipment, and entailed lower management costs. However, they required large testing spaces and long performance sessions, and they were easily influenced by weather. In addition, exercise commonly induces physiological stress, but these tests did not monitor physiological variables. As a result, it was difficult to identify whether the participant reached a state of willpower failure, which is prone to increasing the risk of sudden cardiac arrest in participants with low levels of physical fitness. This would also affect to some extent the effectiveness of $\dot{V}$O_2max_ prediction equations and limit the feasibility of testing in home settings.

To overcome the time and space limitations in CRF field-based exercise tests, several researchers proposed the use of step-up tests to evaluate CRF levels in adults [14,15,16,17]. Based on the relationship between oxygen consumption in the human body and the post-exercise heart rate (HR) during recovery, they established the $\dot{V}$O_2max_ prediction equation with a combination of demographic parameters (such as age-, gender-, and body-fat-related values) to access the CRF levels of participants, and the outcomes were considerable. The step-up test is one of the most commonly used indirect methods of measurement for estimating CRF [14,16,17]. This type of method requires little space, a short testing time, and no expensive equipment or professionals to operate it. However, some studies found that, during the step-up test, elderly adults and obese individuals were not able to satisfactorily complete the testing procedure at a standard intensity of exercise [6,18]. The step-up test is performed with a step-box with a height of 20–50 cm, so individual differences in fitness level could easily be ignored. For a participant who is overweight or has a knee injury, gait abnormality, or balance impairment, it may be difficult to complete the test.

The step-in-place test may be a suitable alternative to the step-up test. The step-in-place test requires less testing time, space, and equipment. Since it requires no step-box, it is safer than the step-up test. It is also easier to manage and perform in home settings. According to the step-in-place test protocol, the participant lifts the knees to a target height, defined as midway between his/her midpoint of the patella and iliac crest, while standing [2,19]. Currently, the step-in-place test is widely applied in the assessment of aerobic fitness in elderly people (aged 60–94 years), but few studies have examined CRF tests in younger adults. To enhance the safety, effectiveness, and universality of adult CRF tests, with selective and economic considerations, we aimed to develop the 3 min incremental step-in-place (3MISP) testing method and $\dot{V}$O_2max_ prediction equations based on different situations. The main purposes of this research were to analyze the relationship between actual measured values of $\dot{V}$O_2max_ and exercise HR during the 3MISP test and, along with anthropometric parameters, to establish the $\dot{V}$O_2max_ prediction equation, as well as to verify and compare the validities of different $\dot{V}$O_2max_ prediction models. In this research, we hypothesized that variation in exercise HR during the 3MISP test is a potential predictor of $\dot{V}$O_2max_. The $\dot{V}$O_2max_ prediction equations, established using 3MISP-HR variables and combining age, gender, and percent body fat (PBF)/body mass index (BMI), can improve the accuracy of $\dot{V}$O_2max_ prediction.

## 2. Materials and Methods

### 2.1. Study Design

In this research, the participants were required to complete 2 exercise tests: direct measurement of $\dot{V}$O_2max_ and the 3MISP test. An electromagnetically braked bicycle ergometer (Excalibur Sport Ergometer, Lode BV, Groningen, The Netherlands) was used in combination with the Cardiopulmonary Exercise Testing System (Vmax Encore 29 System, VIASYS Healthcare Inc., Yorba Linda, CA, USA) to measure the $\dot{V}$O_2max_ in both the training group and the testing group, while the Polar H10 Heart Rate Monitor with a chest strap (Polar Electro Oy, Espoo, Finland) was used to measure the HR of each participant during the 3MISP test. Due to the significant correlation between the variation in HR during exercise and measured $\dot{V}$O_2max_ [2,3], HR was treated as a predictor of $\dot{V}$O_2max_ to improve the accuracy of $\dot{V}$O_2max_ prediction. With the measured data, this research established multiple linear regression equations based on the parameters of age, gender, PBF/BMI, and with or without 3MISP-HR to predict $\dot{V}$O_2max_. Subsequently, the predicted residual error sum of squares (PRESS) and constant error (CE) statistical methods were adopted separately to cross-validate these prediction equations. These study procedures were approved by the Institutional Review Board of the Industrial Technology Research Institute. Before beginning the experimental tests, informed consent forms were completed by the participants.

### 2.2. Participants

All the participants (Taiwanese adults) were recruited openly, independently, and randomly through public advertisements posted in public spaces. Participants with cardiovascular, pulmonary, or metabolic disorders, or muscular or bone diseases that could affect their completion of the exercise tests, were excluded. Finally, a total of 205 healthy adults (aged 20–64 years, 48.8% women and 51.2% men) completed this research project. The anthropometric and body composition parameters that were measured included height, body weight, BMI, and PBF. A body composition analyzer (InBody ^®^ 570, Biospace, Inc., Seoul, Korea) was used for the measurements of body weight and PBF [20]. BMI was calculated as weight (in kilograms) divided by height (in meters) squared.

### 2.3. Maximal Oxygen Consumption

The $\dot{V}$O_2max_ of each participant was measured with an electromagnetically braked bicycle ergometer and a cardiopulmonary exercise testing system. During the exercise test, the participant was required to wear a Polar H10 Hear Rate Monitor with a chest strap to monitor his/her HR and an appropriate gas-collecting mask (Hans Rudolph). The sampling tube and digital flow sensor connected to the mask were used to measure the tidal volume of each breath and the composition of O_2_ and CO_2_. The initial load at the beginning of the test on the bicycle ergometer was 25 W. The load was increased by 15 W every 2 min until the participant could not maintain the required pedaling rate of 70 rpm. Participants were then asked to rate their levels of physical fatigue on the Borg Rating of Perceived Exertion scale (RPE, scale range: 6–20 points). In the present research, $\dot{V}$O_2max_ refers to the maximum average relative $\dot{V}$O_2_ value for 30 consecutive seconds. The $\dot{V}$O_2max_ criteria were deemed to be met when the participants reached three of the following four requirements: (1) a plateau in $\dot{V}$O_2_ despite an increase in load; (2) respiratory exchange ratio ≥ 1.10; (3) maximum HR over 90% of the age-predicted maximum HR (i.e., 220 − age); and (4) RPE ≥ 18 [21].

### 2.4. MISP Test

Before the 3MISP test, the participant was required to wear a Polar H10 Hear Rate Monitor with a chest strap to monitor his/her HR during exercise. While wearing the HR monitor, the patient stood while the midway point between the participant’s patella and iliac crest was measured as the target height for lifting the knees and marked by colored tape. Once the test began, the participant was asked to match a rhythm produced by an electronic metronome while stepping in place, raising the knee to the marked height with each step. The 3MISP test started with 96 steps per minute (SPM), and the rate was increased by 24 SPM every 1 minute. If the participant was unable to maintain the rhythm, he/she could run instead of walking for up to 3 min. If the participant was unable to lift the knees to the required height or follow the rhythm for 30 s, then the test session was terminated and the results were eliminated from the analysis. For safety concerns, the participant had to maintain the step rate at 80 SPM for a cool-down period of 30 s before resting in a standing position. The recorded data contained the HR at the beginning of exercise (HR0); at the first (HR1), second (HR2), and third minutes (HR3) during the exercise; and at the first minute post exercise (HR4).

### 2.5. Statistical Analysis

All values are presented as mean ± standard deviation (SD). Multivariate analysis of variance was used to compare the differences in physical characteristics between the training and testing groups. The effect size was calculated to reflect the magnitude of between-group differences in the total values for various variables, using Cohen’s d [22].Pearson’s correlation coefficients were calculated to analyze the linear relationships between independent variables (i.e., age, gender, PBF, BMI, and 3MISP-HR) and the measured $\dot{V}$O_2max_ of the training group, and the validity of $\dot{V}$O_2max_ prediction models was also verified. For absolute values of the correlation coefficient (r), 0.00–0.10 is regarded as negligible, 0.10–0.39 as weak, 0.40–0.69 as moderate, 0.70–0.89 as strong, and 0.90–1.00 as very strong correlation [23]. Multiple linear regression analysis with cross-validation (70% of the samples were used for modeling, and 30% of the samples were used for verification) was applied to the development of $\dot{V}$O_2max_ prediction models by using the variables of age, gender, PBF/BMI, and with or without 3MISP-HR. The multiple coefficient of determination (R^2^), the absolute SEE, and relative SEE (%SEE) were used for evaluating the accuracy of the $\dot{V}$O_2max_ prediction equation, while the paired t-test was used to compare the difference between the measured $\dot{V}$O_2max_ and estimated $\dot{V}$O_2max_ in the training group. The PRESS and constant error (CE = ∑(Y − Ŷ)/N, where Y is measured $\dot{V}$O_2max_ and Ŷ is estimated $\dot{V}$O_2max_) statistical methods were adopted separately to cross-validate the $\dot{V}$O_2max_ prediction models [3,24]. According to the CRF classifications ($\dot{V}$O_2max_) from the American College of Sports Medicine’s guidelines for exercise testing and prescription, the entire sample was divided into the subgroups of gender, age, and $\dot{V}$O_2max_ [21], and then the CEs were calculated to compare the differences between the measured and estimated $\dot{V}$O_2max_ among these subgroups. Bland–Altman plots were applied to assess the agreement between the predicted and directly measured $\dot{V}$O_2max_ values [25]. The statistical software SPSS (version 22, IBM Corp., Armonk, NY, USA) was used for statistical analysis. The significance level was set to *p* < 0.05.

## 3. Results

Table 1 presents the study population and the physical characteristics of all participants in the training and testing groups. The results of multivariate analysis of variance showed that there were no significant differences in age, height, body weight, BMI, PBF, HR0, or ΔHR3 − HR4 between the training and testing groups. Average $\dot{V}$O_2max_ was higher in the training group than in the testing group.

Table 2 presents the Pearson’s correlations between measured $\dot{V}$O_2max_ and independent variables in the training group. The results showed that age (r = −0.239, *p* = 0.004), BMI (r = −0.191, *p* = 0.022), PBF (r = −0.706, *p* < 0.001), and HR0 (r = −0.516, *p* < 0.001) all had significant negative correlations with $\dot{V}$O_2max_, while gender (women = 0, men = 1) and ΔHR3 − HR4 both had positive correlations with $\dot{V}$O_2max_ (gender: r = 0.430, *p* < 0.001; ΔHR3 − HR4: r = 0.563, *p* < 0.001).

**Figure 1:**
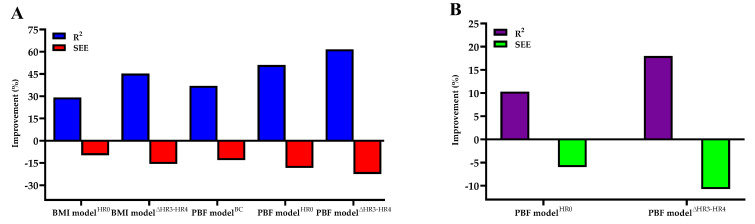
(**A**) Percentage improvement in $\dot{V}$O_2max_ prediction accuracy in the BMI model^HR0^, BMI model^∆HR3 − HR4^, PBF model^BC^, PBF model^HR0^, and PBF model^∆HR3 − HR4^ as compared with the BMI model^BC^. (**B**) Percentage improvement of $\dot{V}$O_2max_ prediction accuracy in PBF model^HR0^ and PBF model^∆HR3−HR4^ as compared with the PBF model^BC^.

Table 3 lists the multiple regression models for predicting $\dot{V}$O_2max_ and the results of cross-validation. Among the PBF and BMI models, the PBF model^∆HR3−HR4^ had the highest multivariate correlation and the lowest SEE value. When age, gender, and body composition were used to predict $\dot{V}$O_2max_, the addition of HR0 increased R^2^ from 0.545 to 0.601 and decreased the SEE from 4.7757 to 4.4905 mL·kg^−1^·min^−1^ in the PBF model^HR0^, whereas in the BMI model^HR0^, R^2^ increased from 0.398 to 0.514 and the SEE decreased from 5.4936 to 4.9564 mL·kg^−1^·min^−1^. Therefore, the variance of $\dot{V}$O_2max_ explained by the PBF and BMI models^HR0^ increased by 10.28% and 29.15%, respectively, while the SEE decreased by 5.97% and 9.78%, respectively. The addition of HR0 and ∆HR3−HR4 increased the explained variance of $\dot{V}$O_2max_ by 17.98% in the PBF model^∆HR3 − HR4^ and 45.23% in the BMI model^∆HR3 − HR4^, while the SEE decreased by 10.73% in the PBF model^∆HR3 − HR4^ and 15.61% in the BMI model^∆HR3 − HR4^ (see Figure 1). The cross-validation results of the PRESS method showed that the changes in R^2^ and SEE values of all $\dot{V}$O_2max_ prediction models were minor (∆R^2^ < 0.014, ∆SEE < 0.2125 mL·kg^−1^·min^−1^).

**Figure 2:**
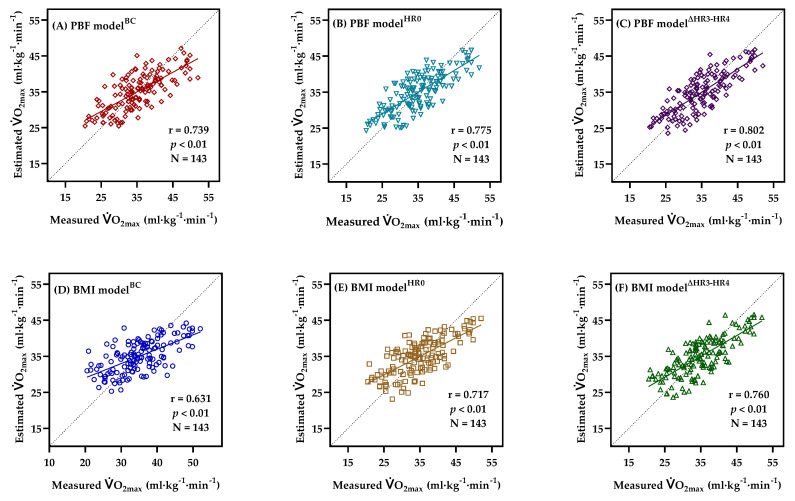
Correlations between the measured and estimated $\dot{V}$O_2max_ obtained from PBF model^BC^ (**A**), PBF model^HR0^ (**B**), PBF model^∆HR3 − HR4^ (**C**), BMI model^BC^ (**D**), BMI model^HR0^ (**E**), BMI model^∆HR3 − HR4^ (**F**), in the training group, showing the regression line and Pearson’s correlation coefficient.

Figure 2 explains the correlation between the measured $\dot{V}$O_2max_ and estimated $\dot{V}$O_2max_ in the training group. There was no significant difference between the estimated $\dot{V}$O_2max_ (PBF model^BC^: 34.96 ± 5.18 mL·kg^−1^·min^−1^; BMI model^BC^: 34.96 ± 4.42 mL·kg^−1^·min^−1^; PBF model^HR0^: 34.96 ± 5.43 mL·kg^−1^·min^−1^; BMI model^HR0^: 34.96 ± 5.02 mL·kg^−1^·min^−1^; PBF model^∆HR3^^−^^HR4^: 34.96 ± 5.62 mL·kg^−1^·min^−1^; BMI model^∆HR3^^−^^HR4^: 34.96 ± 5.33 mL·kg^−1^·min^−1^) and the measured $\dot{V}$O_2max_ (34.96 ± 7.01 mL·kg^−1^·min^−1^). 

**Figure 3:**
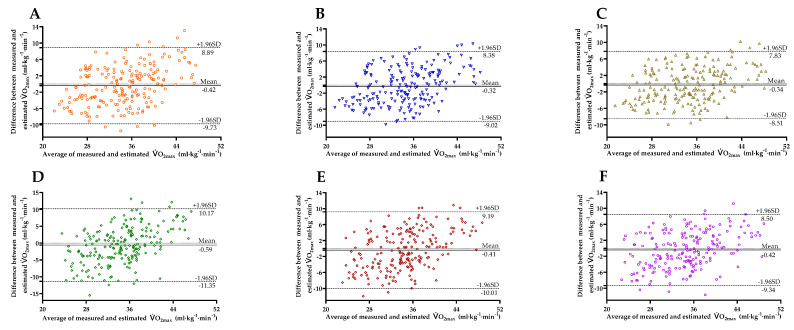
Bland–Altman Plots comparing the differences between measured and estimated $\dot{V}$O_2max_ obtained from PBF model^BC^ (**A**), PBF model^HR0^ (**B**), PBF model^∆HR3 − HR4^ (**C**), BMI model^BC^ (**D**), BMI model^HR0^ (**E**), BMI model^∆HR3 − HR4^ (**F**) in the entire sample (*n* = 205). The mean differences and 95% limits of agreement are shown as solid lines and dashed lines, respectively.

Figure 3 shows the Bland–Altman Plots comparing the values of $\dot{V}$O_2max_ measured with those predicted, with the 95% limits of agreement (LoAs). The mean differences between $\dot{V}$O_2max_ measured and estimated by PBF model^BC^ (95% LoA = −9.73 to 8.89), PBF model^HR0^ (95% LoA = −9.02 to 8.38), PBF model^∆HR3^ ^− HR4^ (95% LoA = −8.51 to 7.83), BMI model^BC^ (95% LoA = −11.35 to 10.17), BMI model^HR0^ (95% LoA = −10.01 to 9.19), and BMI model^∆HR3^ ^− HR4^ (95% LoA = −9.34 to 8.50) were −0.42 ± 4.75, −0.32 ± 4.44, −0.34 ± 4.17, −0.59 ± 5.49, −0.41 ± 4.90, and −0.42 ± 4.55 mL·kg^−1^·min^−1^, respectively, not significant (all *p* > 0.05), and within the acceptable range [26].

Table 4 shows the results of cross-validation with the CE statistical method in the $\dot{V}$O_2max_ prediction models. The CE absolute values for the subgroups of gender, age, and $\dot{V}$O_2max_ of 32–38 mL·kg^−1^·min^−1^ in the PBF and BMI models^∆HR3 − HR4^ were less than 1. As for the $\dot{V}$O_2max_ subgroup, the CE absolute values for the subgroups of low physical fitness ($\dot{V}$O_2max_ < 32 mL·kg^−1^·min^−1^) and high physical fitness ($\dot{V}$O_2max_ ≥ 38 mL·kg^−1^·min^−1^) were higher, while the subgroup of moderate fitness ($\dot{V}$O_2max_ = 32–38 mL·kg^−1^·min^−1^) had lower CE absolute values in all models.

## 4. Discussion

Previous studies have indicated that CRF is closely related to coronary heart disease, all-cause mortality [4,27,28], and COVID-19 mortality [29]. Individuals with high CRF levels have lower risk of dying from COVID-19, while a low CRF level is likely to increase the risk of cardiovascular disease and mortality [30,31]. For a CRF assessment, $\dot{V}$O_2max_ is generally considered as an indicator of CRF level. It can be used for clinical-related classification, such as risk stratification for patients with COVID-19 [32]. However, given the rapid spread of the severe COVID-19, the conventional method used to directly measure the $\dot{V}$O_2max_ on a treadmill or bicycle ergometer in the laboratory is not feasible. Therefore, it is necessary to develop a simple and reliable home testing method to indirectly measure $\dot{V}$O_2max_. Under such circumstances, this research developed the 3MISP testing method and established six $\dot{V}$O_2max_ prediction equations based on physical characteristics, base HR, and exercise test parameters. The validity of different models for $\dot{V}$O_2max_ prediction was verified and compared as well. This research found a significant correlation between 3MISP-HR and $\dot{V}$O_2max_, as hypothesized. The $\dot{V}$O_2max_ prediction equations using age, gender, and PBF/BMI (i.e., PBF and BMI models^BC^) are relatively simple but less accurate. Although the $\dot{V}$O_2max_ prediction equations with baseline HR (i.e., PBF and BMI models^HR0^) can effectively improve the accuracy of $\dot{V}$O_2max_ prediction, the $\dot{V}$O_2max_ prediction equations built from the 3MISP exercise test (i.e., PBF and BMI models^∆HR3 − HR4^) have the highest accuracy. In addition, PBF is a better predictor than BMI. Compared with BMI models, the three PBF models established with physical characteristics, baseline HR, and exercise test parameters present higher R^2^ and lower SEE (%SEE) values. Of the PBF models, the PBF model^∆HR3−HR4^ is the best one for predicting $\dot{V}$O_2max_ and can provide more precise estimation of $\dot{V}$O_2max_ in healthy adults. However, BMI models are more economical and affordable. Individuals can select the corresponding $\dot{V}$O_2max_ prediction equation based on their own conditions and circumstances to evaluate their CRF levels. Due to the simple movement, minimal space and equipment, short testing time, high safety index, and reliability, the 3MISP test is convenient for everyone to conduct CRF self-monitoring at home.

Generally, HR can reflect an individual’s physical fitness and exercise intensity. In a standard CRF test, the HR of a non-athlete is normally close to the age-predicted maximum HR [33]. An individual with a higher CRF level has a lower baseline HR and shorter HR recovery time following a cardiopulmonary exercise test [34]. Since there is a linear relationship between HR variations before, during, and after exercise and $\dot{V}$O_2max_ [2,3,6,17,35,36,37,38], the exercise HR test can improve the prediction of the $\dot{V}$O_2max_ model. Previous studies have demonstrated a significant negative correlation between post-exercise recovery HR and $\dot{V}$O_2max_ [6,36,37,39], and it is an important factor in $\dot{V}$O_2max_ prediction. Matsuo et al. (2020b) indicated that HR both during and following exercise had negative correlations with $\dot{V}$O_2max_, and the HR index composed of these two had the largest correlation with $\dot{V}$O_2max_ [3]. Chung et al. (2021) found that the difference between HR at the third minute during the exercise and recovery HR at the first minute post exercise in the 3 min step test had positive correlations with $\dot{V}$O_2max_ [2]. The results of the present research are consistent with those of previous studies, demonstrating that ∆HR3 − HR4 and $\dot{V}$O_2max_ have a significant positive correlation in the 3MISP test, while HR0 has a negative correlation with $\dot{V}$O_2max_ (see Figure 1). These findings shows that HR can play a potential role in predicting $\dot{V}$O_2max_, and the HR variations based on the 3MISP test can be deemed as one of the relevant factors in CRF for adults. By monitoring the HR response in the 3MISP test, we can objectively understand the physical load during exercise of each participant and further establish the $\dot{V}$O_2max_ prediction equation.

Previous studies indicated that age, gender, and physical characteristics (BMI or PBF) are important predictors of $\dot{V}$O_2max_ [3,17,40]. Those findings are similar to the results of this research. In the present research, the simplest $\dot{V}$O_2max_ prediction equation established with age, gender, and BMI/PBF explained 39.8% of the $\dot{V}$O_2max_ in the BMI model^BC^ and 54.5% of that in the PBF model^BC^ (Table 3). To enhance the accuracy of $\dot{V}$O_2max_ prediction, it uses HR variations during the 3MISP test as the predictive variable to establish the $\dot{V}$O_2max_ prediction equation. The addition of HR0 and ∆HR3 − HR4 increases the variance of $\dot{V}$O_2max_ explained by the PBF and BMI models^∆HR3 − HR4^ by 17.98% and 45.23%, respectively, while SEE decreases by 10.73% and 15.61%. Compared to the most economical BMI model^BC^, the PBF model^∆HR3 − HR4^ increases the explained variance in $\dot{V}$O_2max_ by 61.56%, while the SEE decreases by 22.40% (see Figure 2). These results show that, in the models developed on the basis of biological data, adding 3MISP-HR data improves the accuracy of $\dot{V}$O_2max_ prediction models, and the $\dot{V}$O_2max_ prediction of the PBF model^∆HR3 − HR^ has higher precision than that of the BMI model^∆HR3 − HR4^. Many previous studies of $\dot{V}$O_2max_ prediction have also found that PBF is a better predictor than BMI [2,40,41]. Therefore, when financial conditions permit, people can consider adopting the PBF models to evaluate their $\dot{V}$O_2max_. As for economical options, the BMI models may be affordable choices.

Due to the convenience of the step test, many studies in the past have adopted the Young Men’s Christian Association step test [6,16], Harvard step test [42], Chester step test [42], and Japan step test [3] to evaluate $\dot{V}$O_2max_, with positive outcomes. Lee et al. (2019) established the $\dot{V}$O_2max_ prediction equation based on age, gender, height, body weight, and recovery HR (R^2^ = 0.56–0.61, SEE = 4.74–5.01 mL·kg^−1^·min^−1^) [17]. Hong et al. (2019) used age, sex, body weight, and recovery HR to establish two $\dot{V}$O_2max_ prediction equations, which could explain 73.4% and 72.2% of $\dot{V}$O_2max_, respectively, and the SEEs were both 4.7 mL·kg^−1^·min^−1^ [1]. Matsuo et al. (2020b) used age, gender, BMI, and HR index to establish a $\dot{V}$O_2max_ prediction equation, and the R^2^ and SEE values were 0.60 and 4.05 mL·kg^−1^·min^−1^, respectively [3]. The step-up test requires the participant to continuously step forward onto and backward off a box of a specified height at a speed set by a metronome for a certain time to examine the CRF level according to the HR during or following exercise [43,44]. Since this type of test places high demands on lower limb muscle strength, body coordination, and balance, it is difficult for participants with poor physical fitness or knee injuries to complete, and such participants are likely to fall during the stepping process [2]. Bohannon et al. (2015) noticed that 23% of their participants were unable to complete the step-up test, and those who could complete the procedure were younger adults (aged 39.9 ± 19.4 years) with lower BMI (25.0 vs. 27.1 kg/m^2^) [18]. Beutner et al. (2015) also found that the participants who were unable to complete the test (15%) were older adults (aged 69.3 ± 5.5 years) or had higher BMI (BMI: 29.5 ± 3.9 kg/m^2^) [6]. To enhance the safety, effectiveness, and universality of the CRF assessment, this research designed a substitute for the step-up test, namely the 3MISP test. The midway point between the participant’s patella and iliac crest is measured as the target height for lifting the knees during the test, and no step-box is used. It is thus safer than the step-up test. As compared with previous studies that assessed $\dot{V}$O_2max_ with a step-up test, the R^2^ and SEE values (R^2^ = 0.578–0.643; SEE = 4.2631–4.6358 mL·kg^−1^·min^−1^) in our $\dot{V}$O_2max_ prediction models (PBF and BMI model^∆HR3−HR4^) developed by the 3MISP test are both acceptable. Our study agreed with previous reports suggesting that there was a strong correlation between $\dot{V}$O_2max_ values predicted using gender, age, physical characteristics (BMI/PBF) and HR from the step test, and actual measurements of $\dot{V}$O_2max_ in the bicycle ergometer or treadmill test [2,3,16] and confirmed the applicability of 3MISP test to the healthy adults.

For determining the reliability and validity of our $\dot{V}$O_2max_ prediction equations, two separate cross-validation procedures were performed in this research. The cross-validation results of the PRESS method showed that the R^2^ (0.002–0.014) and SEE (0.018–0.2125 mL·kg^−1^·min^−1^) differences in the multiple linear regressions for the six prediction models between the training and testing groups were minor (see Table 3). Cross-validation analysis with the CE statistical method was used to compare the difference between measured and estimated $\dot{V}$O_2max_ in subgroups, and the results showed that the CE absolute values for the subgroups of gender, age, and $\dot{V}$O_2max_ of 32–38 mL·kg^−1^·min^−1^ in the PBF and BMI models^∆HR3 − HR4^ were minor (see Table 4). The results of these two separate cross-validations verified the effectiveness of our $\dot{V}$O_2max_ prediction models. Therefore, the proposed 3MISP test and the $\dot{V}$O_2max_ prediction equations established in the present research are reasonable and feasible according to these experimental results.

The practical implications are that HR during the 3MISP test can be used to predict $\dot{V}$O_2max_, providing the assessment of CRF. By developing the $\dot{V}$O_2max_ models, the 3MISP test provides HR making it cost-effective and space-efficient to evaluate the CRF levels. The 3 min Harvard step was applied to measure the CRF levels in Taiwan, by calculating the step-up index with HR. This test requires step-up boxes, and participants with low physical fitness or knee injuries are prone to falling in the process of stepping forward onto and backward off the box [2]. Therefore, the safer test is pursued. Given that the HR is the key indicator of CRF, the implementation of the 3MISP test to calculate HR0 and ∆HR3 − HR4 provides a more securely practical method of CRF measurement, without a step-up box in the present study. Furthermore, we established six $\dot{V}$O_2max_ prediction models, and the public can choose the corresponding formulas based on economic conditions. Under specific conditions, individuals without a PBF detector can select more economical BMI models to estimate $\dot{V}$O_2max_, thereby promoting the self-health management.

There are some strengths and limitations in this research. The strengths are that the 3MISP test proposed for evaluating CRF is simple, safe, effective, space-saving, and easy to conduct. The cross-validation design was used to investigate the validity of 3MISP in predicting $\dot{V}$O_2max_. In addition, the six prediction models can be chosen under different situations. Regarding limitations, no participants were given any habituation trial before the 3MISP test. Another limitation is that, since the participants in this research were all healthy adults in Taiwan aged 20–64 years, the $\dot{V}$O_2max_ prediction equation established here may not be applicable to children, adolescents, older adults, or individuals with metabolic syndrome or mobility impairments. 

## 5. Conclusions

The 3 min incremental step-in-place test has simple movements, a minimal testing space, a short testing time, a high safety index, and reliability, and it requires no step-box or expensive equipment. High demands on rhythm and coordination are limitations of 3 min incremental step-in-place test. This study showed that the 3 min incremental step-in-place test is an effective assessment method, and the accuracy of regression models for predicting maximal oxygen uptake was also improved. Among the six maximal oxygen uptake prediction models developed in the present research, the percent body fat model^∆HR3 − HR4^ using age, gender, percent body fat, and heart rate at the beginning of exercise, and difference between heart rate at the third minute during the exercise and the first minute post exercise in the 3 min incremental step-in-place test, had the highest accuracy and appears to be the best model for maximal oxygen uptake prediction, whereas the body mass index models are more economical and affordable. Individuals can select appropriate maximal oxygen uptake prediction equations based on their own conditions and circumstances to evaluate their cardiorespiratory fitness levels. The 3 min incremental step-in-place test provides a safe, simple, and effective method of assessing cardiorespiratory fitness. It can be applied in the home setting as a cardiorespiratory fitness self-monitoring method for the general population. In the event that a rapid test is required and space is limited, the 3 min incremental step-in-place test can also be used as an ideal choice for clinical practice. The achievements of this study can provide health groups aged 20–64 years with various choices of cardiorespiratory fitness assessment, regardless of whether they own the necessary equipment. 

## Figures and Tables

**Table 1:** Physical characteristics of the participants.

	Training Group			Testing Group			ES
	Women (*n* = 69)	Men (*n* = 74)	Total (*n* = 143)	Women (*n* = 31)	Men (*n* = 31)	Total (*n* = 62)	
Age (years)	43.36 ± 9.51	43.24 ± 10.52	43.30 ± 10.01	44.94 ± 10.36	43.52 ± 10.13	44.23 ± 10.19	−0.09
Height (cm)	160.30 ± 4.95	171.78 ± 5.48	166.24 ± 7.77	159.74 ± 5.50	173.40 ± 6.66	166.57 ± 9.17	−0.04
Body weight (kg)	59.40 ± 8.41	75.57 ± 10.72	67.77 ± 12.60	59.47 ± 10.59	75.90 ± 11.78	67.69 ± 13.86	0.01
BMI (kg/m^2^)	23.08 ± 2.74	25.61 ± 3.32	24.39 ± 3.30	23.22 ± 3.24	25.22 ± 3.50	24.22 ± 3.49	0.05
PBF (%)	28.94 ± 6.25	22.32 ± 6.43	25.51 ± 7.14	29.79 ± 6.20	23.06 ± 5.59	26.42 ± 6.77	−0.13
$\dot{V}$O_2max_ (mL·kg^−1^·min^−1^)	31.85 ± 5.70	37.85 ± 6.90	34.96 ± 7.01	29.40 ± 5.54	36.31 ± 6.42	32.86 ± 6.89 *	0.30
HR0 (bpm)	85 ± 12	80 ± 12	82 ± 12	86 ± 9	84 ± 9	85 ± 9	−0.28
ΔHR3 − HR4 (bpm)	28 ± 8	31 ± 8	29 ± 8	26 ± 8	30 ± 7	28 ± 8	0.13

Notes: Values are presented as mean ± standard deviation (SD). PBF, percent body fat; BMI, body mass index; HR0, heart rate at the beginning of 3MISP exercise; ΔHR3−HR4, difference between heart rate at the third minute during the exercise and the first minute post exercise during the 3MISP test; ES, effect size; * Significant difference (*p* < 0.05) in $\dot{V}$O_2max_ between the training and testing groups in the total values.

**Table 2:** Pearson correlation coefficients for the correlations between $\dot{V}$O_2max_ and independent variables in the training group.

	$\dot{V}$O_2max_	Age	Gender	BMI	PBF	HR0	HR3	∆HR3 − HR4
Age	−0.239 **							
Gender (women = 0, men = 1)	0.430 **	−0.006						
BMI	−0.191 *	−0.044	0.385 **					
PBF	−0.706 **	0.084	−0.465 **	0.334 **				
HR0	−0.516 **	0.007	−0.203 *	0.170 *	0.439 **			
HR3	−0.198 *	−0.177 *	−0.184 *	0.007	0.198 *	0.562 **		
∆HR3 − HR4	0.563 **	−0.102	0.187 *	−0.128	−0.406 **	−0.551 **	−0.116	
HR_peak_	0.308 **	−0.676 **	0.015	−0.057	−0.190 *	−0.065	0.061	0.064

Notes: BMI, body mass index; PBF, percent body fat; HR0, heart rate at the beginning of 3MISP exercise; HR3, heart rate at the third minute during the 3MISP test; ΔHR3 − HR4, difference between heart rate at the third minute during the exercise and the first minute post exercise during the 3MISP test; HR_peak_, peak heart rate during the $\dot{V}$O_2max_ test; * *p* < 0.05; ** *p* < 0.01.

**Table 3:** Multiple regression models predicting $\dot{V}$O_2max_ (mL·kg^−1^·min^−1^).

$\dot{V}$O_2max_ (mL·kg^−1^·min^−1^)	PBF Model (%)			*p* Value	BMI Model (kg·m^−2^)			*p* Value
	B	Standard Error	*β*		B	Standard Error	*β*	
Model^BC^								
Constant	55.261	2.484		<0.001	60.719	4.163		<0.001
Age (years)	−0.130	0.040	−0.186	0.002	−0.178	0.046	−0.255	<0.001
Gender (women = 0, men = 1)	1.925	0.903	0.138	0.035	8.299	0.996	0.594	<0.001
Body composition	−0.614	0.064	−0.626	<0.001	−0.916	0.152	−0.431	<0.001
*F*	55.583				30.686			
*p*	<0.001				<0.001			
R^2^	0.545				0.398			
Adjusted R^2^	0.536				0.385			
SEE (mL·kg^−1^·min^−1^)	4.7757				5.4936			
SEE%	13.662				15.716			
R^2^*p*	0.534				0.396			
SEE*p*	4.8772				5.7061			
Model^HR0^								
Constant	65.240	3.262		<0.001	73.265	4.348		<0.001
Age (years)	−0.135	0.038	−0.194	<0.001	−0.174	0.042	−0.248	<0.001
Gender (0 = women, 1 = men)	1.932	0.849	0.138	0.024	6.707	0.941	0.480	<0.001
Body composition	−0.500	0.065	−0.510	<0.001	−0.691	0.142	−0.325	<0.001
HR0 (bpm)	−0.154	0.035	−0.262	<0.001	−0.212	0.037	−0.361	<0.001
*F*	51.952				36.465			
*p*	<0.001				<0.001			
R^2^	0.601				0.514			
Adjusted R^2^	0.589				0.500			
SEE (mL·kg^−1^·min^−1^)	4.4905				4.9564			
SEE%	12.847				14.179			
R^2^*p*	0.587				0.507			
SEE*p*	4.5226				4.9852			
Model^∆HR3−HR4^								
Constant	51.312	4.650		<0.001	55.761	5.596		<0.001
Age (years)	−0.121	0.036	−0.173	0.001	−0.152	0.039	−0.217	<0.001
Gender (0 = women, 1 = men)	1.927	0.806	0.138	0.018	6.204	0.887	0.444	<0.001
Body composition	−0.452	0.063	−0.461	<0.001	−0.632	0.134	−0.297	<0.001
HR0 (bpm)	−0.085	0.038	−0.145	0.025	−0.120	0.040	−0.203	0.003
∆HR3 − HR4 (bpm)	0.220	0.055	0.253	<0.001	0.267	0.059	0.308	<0.001
*F*	49.338				37.494			
*p*	<0.001				<0.001			
R^2^	0.643				0.578			
Adjusted R^2^	0.630				0.562			
SEE (mL·kg^−1^·min^−1^)	4.2631				4.6358			
SEE%	12.196				13.262			
R^2^*p*	0.651				0.587			
SEE*p*	4.1861				4.6178			

Notes: B, unstandardized regression weights; *β*, standardized regression weights; BC, body composition; PBF, percent body fat; BMI, body mass index; HR0, heart rate at the beginning of 3MISP exercise; ΔHR3 − HR4, difference between heart rate at the third minute during the exercise and the first minute post exercise in the 3MISP test; SEE, standard error of estimate; SEE%, SEE/mean of measured $\dot{V}$O_2max_ × 100; R^2^*p*, PRESS squared multiple correlation coefficient; SEE*p*, PRESS standard error of estimate.

**Table 4:** Constant error and standard deviations for subgroups of gender, age, and measured $\dot{V}$O_2max_ in the entire sample (*n* = 205).

Subgroup	N (%)	PBF Model^BC^		PBF Model^HR0^		PBF Model^∆HR3^ ^− HR4^		BMI Model^BC^		BMI Model^HR0^		BMI Model^∆HR3^ ^− HR4^	
		CE	SD	CE	SD	CE	SD	CE	SD	CE	SD	CE	SD
Gender													
Women	100 (48.8)	−0.53	4.42	−0.51	4.30	−0.46	4.00	−0.63	5.32	−0.58	4.91	−0.50	4.55
Men	105 (51.2)	−0.31	5.07	−0.13	4.59	−0.23	4.35	−0.55	5.67	−0.24	4.91	−0.35	4.57
Age (years)													
<40	71 (34.6)	−0.35	5.11	−0.28	4.47	−0.37	4.15	−0.39	6.16	−0.28	5.16	−0.39	4.78
40–50	72 (35.1)	−1.30	4.48	−1.10	4.31	−0.96	4.16	−1.33	5.17	−1.05	4.73	−0.91	4.51
≥50	62 (30.2)	0.52	4.52	0.54	4.48	0.42	4.16	0.05	5.00	0.19	4.76	0.10	4.35
$\dot{V}$O_2max_ (mL·kg^−1^·min^−1^)													
<32	73 (35.6)	−3.21	4.00	−2.94	3.73	−2.57	3.53	−4.57	4.57	−3.84	4.07	−3.26	3.97
32–38	75 (36.6)	−1.00	3.86	−0.78	3.80	−0.79	3.86	−0.83	3.67	−0.57	3.79	−0.62	3.84
≥38	57 (27.8)	3.91	3.51	3.64	3.12	3.11	2.99	4.83	3.80	4.21	3.17	3.47	3.14

Notes: BC, body composition; PBF, percent body fat; BMI, body mass index; HR0, heart rate at the beginning of exercise; ΔHR3 − HR4, difference between heart rate at the third minute during the exercise and the first minute post exercise in the 3MISP test.
